# The Impact of Maternal/In Utero Exposure of Novel Tobacco Products on the Offspring Cardiovascular Health

**DOI:** 10.1007/s12012-025-10023-5

**Published:** 2025-06-20

**Authors:** Shelby S. Umphres, Precious O. Badejo, Fadi T. Khasawneh, Fatima Z. Alshbool

**Affiliations:** 1 Department of Pharmacy Practice, Irma Lerma Rangel College of Pharmacy, Texas A&M University, 1010 W Avenue B, Kingsville, TX 78363 USA; 2 Department of Pharmaceutical Sciences, Irma Lerma Rangel College of Pharmacy, Texas A&M University, 1010 W Avenue B, Kingsville, TX 78363 USA

**Keywords:** Cardiovascular disease, E-cigarettes, Maternal/in utero exposure, Novel tobacco products

## Abstract

Novel tobacco products (NTPs) have recently been on the rise appealing to a variety of users, including pregnant women and women of childbearing age, who deem these products to be a safe/safer alternative to traditional smoking. To this end, several studies have made advances toward proving the invalidity of these claims, especially in the context of cardiovascular disease. However, an area that has yet to be extensively explored is maternal/in utero exposure to these devices and the cardiovascular health outcomes on the offspring into their adult life. Herein, our aim is to critically assess the literature to identify and discuss the cardiovascular health risks that the offspring exhibits as a result of in utero exposure to NTPs. These studies have been summarized as a comprehensive review.

## Introduction

Extensive research, spanning several decades, on tobacco smoking has shown that cigarette usage during pregnancy can lead to detrimental effects, some of which could manifest starting before birth (such as fetal death and growth restriction), whereas other issues become evident as early as birth [1, 2]. Smoking during pregnancy, also known as “in utero*/*maternal exposure,” can lead to premature birth, low birth weight, high risk of birth deficiencies, sudden infant death syndrome (SIDS), among others [3–7]. Concerning the cardiovascular health effects, in utero*/*maternal tobacco exposure was found to increase blood pressure (BP) in newborns/kids and predispose them to hypertension in adulthood [8–10]. Indeed, nicotine, which is one of the major constituents of tobacco, is documented to cross the placenta easily, thereby leading to a higher concentration in the fetal circulation compared to the mother’s blood circulation [11, 12]. This is due to the notion of placental accumulation, as the placenta serves as a passage for the entry of nicotine (and other chemicals found in cigarettes) into the fetal compartment, through both passive diffusion and active transport [13, 14]. Since the placenta has limited capacity to metabolize nicotine, this contributes to its higher levels in the fetal circulation compared to mothers [15]. In several studies using animal models, it was discovered that nicotine exposure results in increased risk of cardiovascular disease (CVD) in the exposed offspring later in life (i.e., high BP, dyslipidemia, and increase in vascular tone) [16, 17]. Maternal tobacco use could also cause thickening of the heart wall [18], decrease in high-density lipoproteins [19], as well as restructuring of the coronary arteries [20], in the offspring. As a consequence of evidence-based correlation of the negative impact of tobacco smoking/exposure including under in utero/maternal settings on the general and cardiovascular health, tobacco companies invented and introduced into the market novel tobacco products (NTPs) with the claim that they represent safer alternatives [21, 22]. Some of these products use advanced heating technology to deliver nicotine and/or tobacco, such as electronic cigarettes (e-cigarettes), electronic hookahs (e-hookahs or e-waterpipes), and heat-not-burn (HNB) products [23], while others deliver tobacco without generating aerosol/smoke, such as smokeless tobacco [24]. Because of the appealing designs and numerous flavoring options, these products have become more attractive options for vulnerable populations, including women of childbearing age and pregnant women [25]. E-cigarettes are the most used NTPs during pregnancy with 6.5% of usage, which is even higher than that of cigarettes (5.6%), as depicted in a recent study carried out in the United States (US) [25]. To further support this notion, e-cigarettes are more predominant among adults between the age of 18 and 44 years; an age group in which women are more likely to get pregnant [26]. In addition, a larger percentage of pregnant women using e-cigarettes, with 74.6% reported switching from cigarettes to e-cigarettes during pregnancy, with half of this population believing they were a safe/safer choice [25]; however, this notion is not evidence-based and lacks “credibility.” This belief is attributed to limited evidence/studies due to the recent availability of these products in the market, and the fact that the effects of these products on pregnancy and fetal development/offspring health are not well characterized. Thus, preclinical studies utilizing animal models were conducted to address this knowledge gap and provide quick answers concerning the safety of e-cigarettes during pregnancy. For instance, a recent study in mice linked the exposure to e-cigarette aerosol during pregnancy to fetal neural development/negative health effects [27]. Given that CVD is still the number one killer and cardiovascular function has been shown to be impacted by early life/in utero exposure to many environmental insults [28], it is essential to establish the impact of in utero*/*maternal NTPs usage on the offspring’s cardiovascular health. Evidence of the potential cardiovascular harm of NTPs, especially e-cigarettes, comes from preclinical studies using animal models that mimic human usage during pregnancy, which have shed light on their detrimental health impact on fetuses and children/offspring who are involuntarily exposed. Thus, the aim of this review is to summarize the literature related to in utero*/*maternal NTPs exposure and cardiovascular outcomes in the offspring.

### Prevalence of Novel Tobacco Products (NTPs)

The use of traditional cigarettes has declined rapidly in the last few decades in the US [17, 29], in part due to their documented deleterious health effects [30]. As an offshoot of this decline, the usage rate of NTPs, especially e-cigarettes has increased globally, partly as a result of aggressive advertisement from tobacco companies [31]. Hence, from 2020 to 2021, the Centers for Disease Control and Prevention (CDC) recorded a decline in the prevalence of cigarette smoke, whereas e-cigarette usage increased [19], with highest prevalence found in adults 18–24 years of age [20]. The 2014 Population Assessment of Tobacco and Health (PATH) study examined tobacco use among pregnant women aged 18 years and older and found that cigarettes had the highest usage rate (13.8%), followed by e-cigarettes (4.9%), hookahs (2.5%), cigars (2.3%), with other tobacco products showing the lowest usage (< 1%) [32]. Interestingly, dual usage of e-cigarettes and traditional cigarettes during pregnancy has been on the rise [33], which may be the underlying reason for the high numbers observed in cigarette use. In other studies, e-cigarette usage among pregnant women was found to increase from 2.2% to 3.6% between 2014 and 2018 in the US [34, 35]. Additionally, the use of other tobacco products is on the rise, for example, hookah use during pregnancy ranged from 5.4% to 8.7% [36, 37].

Given that the age of reproduction is considered to be 15–44 years of age [38–40], it is important to highlight the implications of the rise of use among the youth (especially high school students) as well as women of childbearing age. Notably, the most recent report from the National Youth Tobacco Survey (NYTS; 2024) revealed that around 2.25 million youth are currently using tobacco products in the US [41]. Among these, e-cigarettes were the most popular product (5.9%), followed by nicotine pouches (1.8%), cigarettes (1.4%), cigars (1.2%), smokeless tobacco (1.2%), heated tobacco products (0.8%), and hookahs (0.7%) [41]. The fact that these products are gaining popularity among vulnerable populations (youth, women of reproductive age, and pregnant women) is not surprising, given many of these products are marketed as “no harm” or “less harmful” than cigarettes [42]. In addition, the presence of pleasant flavors, which could be particularly appealing during pregnancy due to changes in taste and cravings, seems to have aided their popularity [23]. Thus, we will review the evidence related to the cardiovascular health impact on the offspring who were in utero/maternally exposed to common NTPs, which includes e-cigarette, e-hookah, hookah, and smokeless tobacco.

## The Cardiovascular Effects of Maternal Exposure to Novel Tobacco Products

### E-c igarettes

E-cigarettes are battery operated devices that vaporize e-liquids, which typically consists of propylene glycol (PG), vegetable glycerin (VG), a flavoring component, and varying concentrations of nicotine [43]. E-cigarettes come in a variety of styles and flavored e-liquids, which is also an appealing aspect to the users and considered a major contributor to their rapid growth [43–45]. However, with their recent availability in the market, there has not yet been enough time to establish their long-term effects and provide concrete evidence regarding their safety claims, including under maternal/in utero exposure conditions. Thus, investigation of the potential/deleterious effects these devices can pose on human health is warranted, specifically in the context of CVD as it remains the leading cause of death worldwide [46]. While there is ample evidence concerning the negative effects of e-cigarette use during adulthood on the cardiovascular system [47–50], limited studies and evidence exist connecting the offspring cardiovascular health with maternal e-cigarette use during pregnancy. Therefore, we aim to summarize studies on the cardiovascular effects of maternal/in utero e-cigarette exposure. The limited reports on this topic do not allow categorization by specific CVD, so the literature will be summarized as a whole (Table 1).

**Table 1:** Summary of cardiovascular outcomes in the offspring of maternal NTP and alternative tobacco product users

Novel tobacco product	Experimental model	Exposure duration	Puffing topography	Nicotine concentration	Flavor and VG/PG	Cardiovascular effects	References
E-cigarette	Zebrafish, hESCs	Group 1: 24 h Group 2: 48 h Group 3: 72 h	1 aerosolized e-cigarette cartridge	6.8, 13.7 or 34 µM in vivo 1.7, 3.4. 6.8 or 13.7 µM in vitro	Tobacco classic	↓ Expression of cardiac transcription factors, ↑ incidence of heart defects	[51]
E-cigarette	Zebrafish Embryos	24 h	0.6, 12, or 25 puffs/L, 55 ml puff, 4 s puff duration every 30 s	nicotine free	cinnamon, and chocolate 70/30 PG/VG	↓ HR, EDV, ESV, Q, SV, and RBC density	[54]
E-cigarette	Sprague Dawley rat	Exposure started at gestational day 2 and ending postnatal day 21 (gestation and postnatal)	1 h/day, 5 days/week, 1 puff every 3 min (total 20 puffs) or 1 h/day, 5 days/week, 1 puff every 1 min (total 60 puffs) 5 s puff duration	0 mg/ml or 18 mg/ml	French vanilla 75/25 VG/PG	↑ PWV, impaired aortic relaxation, ↓ elastin, ↑ collagen, ↓ NO bioavailability and vascular dysfunction	[59]
E-c igarette	Sprague Dawley rat	Gestational day 2 to postnatal day 21 (gestation and postnatal)	1 h/day, 5 days/week, 1 puff every 3 min (total 20 puffs) 5 s puff duration	0 mg/ml or 18 mg/ml	French vanilla 75/25 VG/PG	↓ MCA reactivity, ↓ EDD	[66]
E-c igarette	Sprague Dawley rat	Group 1: gestational days 5 to gestation day 20 (Gestation) Group 2: gestational days 5 to gestation day 20 and at postnatal day 4 to 9 (Gestation and postnatal)	3 h/day, 5 days/week, 1 s puff every 20 s or 2 h/day, 5 days/week, 1 s puff every 20 s 42 ml puff	0 mg/ml or 100 mg/ml	No flavor 80/20 PG/VG	↓ Maternal and fetal blood flow	[67]
E-cigarette	Sprague Dawley rat	Group 1: gestational days 2–7 (1st trimester) Group 2: gestational days 8–14 (2nd trimester) Group 3: gestational days 14–21 (3rd trimester) Group 4: gestational days 2–21 (full-term gestation)	90 min/day, 5 days/week, 5-s puff every 90 s (total 60 puffs) 1 or 3 weeks	0 mg/ml	No flavor 50/50 PG/VG	↓ MCA reactivity, ↓ EID and contractile response	[72]
E-cigarette	Sprague Dawley rat	Gestational days 2–21 (gestation)	60 puffs/day, 5 days/week	0 mg/ml	No flavor 50/50 PG/VG	↓ MCA reactivity	[79]
E-cigarette	Sprague–Dawley rat	Group 1: gestational days 2–21 (gestation only) Group 2: 4-week exposure at either 2- or 5-month-old offsprings (Adolescence or adult age) Group 3: gestational days 2–21 And 4-week exposure at either 2 or 5 months old (Gestation and Adolescence/ adult age)	90 min/day, 5 days/week, 5 s puff every 90 s (total 60 puffs) 3 weeks	0 mg/ml	No flavor 50/50 PG/VG	↓ MCA reactivity, ↑ Plasma EV’s, ↑ Oxidative stress	[81]
E-cigarette	Mouse	Gestational days 4–20 (gestation)	12 h/day, 16 days 4 s puff every 30 s	2.4%	Tobacco	↑ cardiomyocyte apoptosis, ↑ cardiac oxidative stress, enlarged mitochondria, apoptotic nuclei, myofibrillar derangement, and convoluted nuclear membranes	[83]
Waterpipe tobacco	Wistar rats	1 day prior to mating until birth (gestation)	2 h/day 2.6 s puff every 17 s (total 171 puffs) 530 ml puff	10 g of Ma’assel	Apple	↑ heart mass in offspring, ↑ heart-to-body mass ratio	[89]
Smokeless tobacco/Swedish snus	Human	Gestational weeks 6–12 until birth	Variable between users	> 48 mg of nicotine daily	Tobacco	↑ systolic BP, ↑ carotid stiffness index, ↑ EDD	[97]
Smokeless tobacco/Swedish snus	Human	Entire pregnancy	Variable between users	Snus exposure ≥ 48 mg/d nicotine dose	Tobacco	↑ mean SPB, ↑LF/ HF (heart rate variability)	[98]

*PWV* pulse wave velocity, *HR* heart rate, *EDV* end-diastolic volume, *ESV* end-systolic volume, *Q* cardiac output, *SV* stroke volume, *RBC* red blood cell density, *NO* nitric oxide, *BP* blood pressure, *EDD* endothelium-dependent dilation, *SPB* systolic blood pressure, *LF* low frequency, *HF* high frequency, *MCA* middle cerebral artery, *EID* endothelial-independent dilation, *EVs* extracellular vesicles, *PG* propylene glycol, *VG* vegetable glycerin, *GD* gestational day

Using a zebrafish model as well as human embryonic stem cells (hESCs), cardiac development in the context of e-cigarette exposure was investigated [51]. Under both in vivo and in vitro settings, Zebrafish or the hESCs were subjected to varying concentrations of e-cigarette aerosol, purified nicotine, and tobacco cigarettes. Of note, the highest concentration of e-cigarette aerosol (34 µM nicotine) induced a significantly lower survival rate of the embryos, post 72 h of exposure, and therefore, the lower doses, 6.8 µM and 13.7 µM of nicotine, were used for the remaining experiments. Heart abnormalities in embryos were assessed and classified into 4 groups based on their severity: normal, mild, intermediate, and severe. Interestingly, the nicotine alone did not display any heart abnormalities that were considered significantly different from the controls; however, e-cigarette exposure did show incidence of heart defects which were significant for both concentrations, categorizing most as mild, and some cases as intermediate and severe. However, tobacco cigarettes resulted in severe heart malfunction, which suggests a higher toxicity than e-cigarette, at least under the present experimental conditions. It was also found that e-cigarette exposure does not produce significant differences in the expression of a panel of genes critical for heart development (e.g., cmlc2, tnnt2, and cx43). Moreover, e-cigarette exposure did not show any significant differences in cardiac immaturity, which was assessed by measuring beating rate, cardiomyocyte yield, cardiomyocyte purity, and the expression of smooth muscle actin. However, a decreased expression of transcription factors and markers of maturation such as MLC2v and β-MHC was observed in e-cigarette treated hESCs, suggesting a delay in differentiation. Although the effects of e-cigarette exposure are not as profound as tobacco cigarettes, it is important to note that it does pose detrimental effects on cardiac development, which is an alarming health concern. Interestingly, the finding that nicotine alone is not different from controls and does not resemble either e-cigarettes or tobacco cigarette effects, seems to suggest negative effects for other e-cigarette/cigarette components in cardiac development under maternal conditions, which is consistent with studies for adult exposure to various e-liquid and e-vapor components, such as VG, PG, and formaldehyde [52, 53]. In addition, studies found that flavors are involved in e-cigarette mediated toxicity under direct exposure conditions [54, 55]. Piechowksi and Bagatto compared the acute cardiovascular effects of two nicotine free flavored e-liquids during developmental stages using a zebrafish embryo model [54]. In this study, the embryos were exposed to three different doses of either cinnamon or chocolate flavored vape, and all cardiovascular parameters were measured 24 h following the exposure. At the highest dose, only the cinnamon flavored vape altered cardiovascular functions and resulted in a significant decrease in end-diastolic volume (65%), end-systolic volume (55%), stroke volume (73%), cardiac output (81%), heart rate (25%), and red blood cell density (80%), when compared to the untreated control. On the other hand, the chocolate flavor did not produce any significant differences relative to the control. Additionally, the lower exposure concentrations were also found not to be significant in either flavor. These results indicate that even in the absence of nicotine, e-cigarettes can suppress vital cardiovascular functions in a flavor- and dose-dependent manner. Though not shown under in utero conditions, e-liquid flavoring components were found to be genotoxic and cytotoxic in vitro [56, 57]. As for the mechanism by which cinnamon flavor leads to these effects, the authors suggest that it partly involves cinnamaldehyde, which has been previously shown to alter cardiovascular functions in an adult mouse model, by inhibiting L-type calcium channels [58]. It is important to note that further studies are warranted in order to address the impact of other e-liquid flavors as well as e-liquid components on other cardiovascular outcomes and disease states and whether some of the outcomes observed would persist in a fully developed heart and post embryonic exposure.

In one recent preclinical study, pregnant Sprague Dawley rats were exposed to e-cigarette vape starting at first day of gestation until weaning, before the long-term effects on arterial function were determined in 3-month and 7-month-old offspring [59]. In this study the investigators exposed rats to nicotine free (0 mg/ml) or nicotine containing (18 mg/ml) e-cigarette vape, and two different numbers of puffs (20 or 60 puffs/day) to address the nicotine-, and dose-dependent effects. Interestingly, the dysfunction of conduit vessel remodeling and conduit artery stiffness, reactivity, and structure were found to be negatively impacted under all the aforementioned exposure conditions (nicotine concentration, puff number, and offspring age) when compared to the controls [59]. The first parameter tested was carotid pulse wave velocity, an indicator of arterial stiffness and CVD [60], which was found to be significantly increased in all exposure groups to the same degree. Of note, while similar results were previously reported with direct e-cigarette exposure in both human and mouse studies [61–63], it is rather more alarming to observe such effects even under in utero conditions. In addition, when histologic staining in the aorta was conducted to assess structural changes, it was observed that the ratio between elastin to collagen was reduced by 41%–54% under all conditions, which is suggestive of an abnormally stiff vessel wall. As far as aortic reactivity and function, aorta relaxation was shown to be impaired in all exposure groups, in response to methacholine. Based on these data, the authors suggested an early onset of hypertension as the contributing factor to these observations, as arterial stiffness has been shown to be increased in the offspring when the parents have an altered hemodynamic state [64]. Additionally, it is well documented that endothelial dysfunction is a risk factor for CVD, which can be linked to reduced nitric oxide (NO) bioavailability [59]. Vaping has been shown to contribute to this imbalance in human studies [65], a notion that was further explored. In order to assess the contribution of NO, the authors utilized the NO inhibitor, nitro-L-arginine methyl ester (L-NAME), and found that endothelial-dependent aortic relaxation was reduced in all experimental groups in its presence, indicating that reduced NO bioavailability is a key factor in the observed aortic dysfunction [59]. Although there are several factors that could influence endothelial dysfunction, vaping-induced cardiovascular alterations could be associated with increased levels of reactive oxygen species (ROS), leading to a state of oxidative stress. Consequently, antioxidant treatments (TEMPOL or febuxostat, superoxide dismutase mimetic or xanthine oxidase inhibitor, respectively) were used, and indeed, restoration of aortic dysfunction was observed, supporting the concept that oxidative stress does contribute to maternal/in utero e-cigarette mediated vascular impairment. Taken together, this study provides evidence that e-cigarette exposure during pregnancy can lead to persistent vascular dysfunction (i.e., increased arterial stiffness and endothelial dysfunction) later in the adult life of the offspring in a nicotine-independent manner. Additionally, since similar results were observed with the 20 and 60 puffs/day, the authors concluded that a low vaping dose can be still harmful to vascular function, thereby underscoring the claim that even low levels of exposure during pregnancy may not be safe (in their rat model). In another study with similar exposure conditions, the cerebrovascular risk was assessed by measuring middle cerebral artery (MCA) reactivity in the offspring at 1, 3, and 7 months of age [66]. It was shown that there was a 51–56% decrease in the endothelial-dependent dilation (EDD), when the MCA was treated with acetylcholine (Ach), for both the 0 mg/ml and 18 mg/ml of nicotine and for all of the three age groups. These findings further support the notion that even without nicotine, in utero e-cigarette exposure poses cerebrovascular risk in the offspring. Blocking NO production with L-NAME blunted Ach-induced MCA reactivity in control animals, but did not significantly alter the already impaired response in the e-cigarette groups, which indicates that reduced NO bioavailability is a major contributing factor to the observed impairment. Interestingly, full restoration of EDD was observed when the MCA was incubated with TEMPOL (an antioxidant), suggesting the contribution of oxidative stress. Of note, endothelial-independent dilation was tested with sodium nitroprusside (SNP), and vasoconstriction in response to 5-hydroxytryptamine (5-HT), but no differences between the exposed and control groups were observed.

Furthermore, and within the theme of vascular dysfunction, a separate study focused on vascular hemodynamics during early development (such as fetal umbilical and maternal uterine blood flow) post-exposure to nicotine (100 mg/ml) or nicotine free aerosolized e-liquid [67]. Oxygen and nutritional requirements of a fetus are generally maintained as the maternal uterine artery circulates blood as an adaptive mechanism to accommodate the needs of the developing fetus [67–71]. The authors determined that only the nicotine containing vape showed a decrease (65.33%) in umbilical artery blood flow and a decrease (49.5%) in uterine artery blood flow. Of note, no differences were observed in maternal and fetal heart rates between any of the groups. Because of the restricted blood flow, growth deficits were also seen in the offspring, further highlighting the influence that the cardiovascular system has on many health and developmental outcomes. Lastly, contrary to some of the aforementioned studies, the nicotine-free vape studies did not show an impact on vascular hemodynamics. However, aside from the different exposure conditions including the use of a very high concentration of nicotine, the fact that this study assessed hemodynamic changes during early development and did not assess the long-term effects in the offsprings’ adulthood life could account for these discrepancies.

While many studies have addressed the effects of in utero exposure in the context of full term [67] or full term in conjunction with lactation [59, 66], the next study focused on the influence of e-cigarette exposure during different gestational periods, specifically in the context of the vascular health of the offspring [72]. Pregnant Sprague Dawley rats were exposed to a nicotine free/no flavor vape at either 5 or 30 watts (W) during the following periods: pre-conception (3 weeks before mating); first trimester (Gestational day (GD) 2–7); second trimester (GD 8–14); third trimester (GD 15–21); and full term (GD 2–21). At 3 months of age, it was reported that the MCA reactivity in response to Ach (endothelial-dependent dilator) was significantly decreased by 18% in the second trimester, 23% in the third trimester, 31% full term (5W), and 43% full term (30W) in the offspring. Interestingly, no significant changes in MCA were observed in the pre-conception exposure group. Additionally, MCA reactivity in response to SNP (endothelial-independent dilator) and contractile response in response to 5-HT (vasoconstrictor) decreased only in the full-term-exposed offspring. Similar results in MCA reactivity in response to Ach, SNP, and 5-HT were observed in the offspring at 6 months. Consistent with previous studies [59, 66, 67], the authors suggested the involvement of endothelial-dependent pathways based on the impaired vascular responses to Ach in the second and third trimester, as well as full-term e-cigarette exposure groups. However, it should be noted that only the full-term exposure group exhibited impaired response to SNP (an endothelial-independent pathway), indicating a potential adverse effect of full gestational period exposure on non-endothelial mechanisms. In addition, the role of NO and oxidative stress was also evaluated using L-NAME (NO synthase inhibitor), TEMPOL (a superoxide dismutase), and febuxostat (a xanthine oxidase inhibitor). In 3-month-old offspring, it was shown that TEMPOL significantly improved the Ach-mediated MCA reactivity response for full-term and third trimester exposure groups in both males and females. Additionally, vasodilation induced by Ach was also decreased following incubation with L-NAME in the first and second trimesters for the males and in the first trimester and full-term (30W) exposure periods for females. Moreover, when the vessels were incubated with febuxostat, sex dependent improvement in MCA reactivity was observed especially in the male full-term exposure groups, but not the females. At 6 months of age, the observed vascular dysfunction was improved with TEMPOL incubation for both second and third trimesters. Ach-mediated dilation was blunted with L-NAME incubation in all groups relative to baselines, but the magnitude of this blunting effect in the full-term (30W) exposure group was statistically significant in comparison to the control group. Lastly, febuxostat restored the MCA reactivity in the third trimester and full-term-exposed male rats while females, once again, displayed no differences. These data suggest that oxidative stress is a potential mechanism for the observed phenotype as it is known to contribute to vascular dysfunction. To this end, it has been reported that production of reactive oxygen species from known chemicals present in e-cigarette aerosols alters/influences cellular processes of the fetus in developmental stages [73–78]. This study collectively suggests that many adverse effects, specifically in the context of vascular dysfunction under in utero exposure conditions, occur in either the second or third trimesters, and that the results are similar to full-term exposure. This interestingly indicates that the critical window that influences vascular dysfunction occurs in the later periods of gestation as opposed to exposure during pre-conception or the first trimester. It is important to highlight that this study utilized e-liquid free of nicotine or flavor, which further supports the notion that the base e-liquid that is composed of VG/PG does also contribute to the adverse vascular outcomes that manifest in the offspring.

In addition to studying critical gestational windows with regard to cardiovascular outcomes, it was also of interest to determine if the lactation period could play a role in postnatal vascular dysfunction [79]. To assess the effects of the lactation period on the offspring, a cross-fostering approach was implemented at birth where half of the clean air and e-cigarette-exposed dams exchanged their litters. Specifically, dams exposed to clean air fostered pups from dams exposed to e-cigarettes and vice versa. For the controls, the remaining half of the dams in each exposure group maintained their own litters. It was found that pups from e-cigarette and e-cigarette cross-fostered with clean air rats during lactation showed a 42–50% lower MCA reactivity in response to Ach at one month of age. However, offspring born from maternal clean air rats that were cross-fostered with those exposed to e-cigarettes did not show any differences in comparison to clean air control. Taken together, these studies suggest that vascular dysfunction observed in in utero e-cigarette-exposed offspring is due to abnormal development during gestation and not lactation. It is important to note that the e-liquid used in this study does not contain nicotine and flavor, thus these results may not be applicable to nicotine/flavor containing e-liquid/e-cigarettes, a notion that warrants further exploration as nicotine is known to cross the blood stream to breast milk [80].

In light of the fact that e-cigarettes are common in youth and early adulthood, a recent study sought to examine the impact of their “direct exposure” in the offspring when combined with a history of maternal/in utero exposure, called “double-hit model,” in the context of vascular function [81]. In this study, Sprague Dawley rats were exposed in 4 different groups, which included (1) maternal and offspring air-exposed rats (Air: Air), (2) maternal e-cigarette- and offspring air-exposed rats (E-cig: Air), (3) maternal air- and offspring e-cigarette-exposed rats (Air: E-cig), and (4) maternal and offspring e-cigarette-exposed rats (E-cig: E-cig; the “double-hit model”). Direct exposure of the offspring started at 2 months or 4 months of age and continued for 1 month, in which experiments were conducted on adolescent rats (3 months of age) and adult rats (6 months of age), respectively. The MCA response to Ach was reduced in all e-cigarette groups which displayed endothelial-dependent impairment; while the “double-hit model” was found to exhibit the highest impairment in both age groups. In contrast, endothelial-independent dysfunction to SNP was only seen in the “double-hit model” at 3 months and in both the “double-hit model” and direct e-cigarette exposure (Air: E-cig) groups at 6 months. Lastly, the contractile response assessed using 5-HT treatment revealed weakened constriction in the group of only maternal e-cigarette exposure; however, an increase in the contractile response was observed in the models where the offspring were directly exposed to e-cigarettes. Of note, there was no difference in the myogenic response between any control and exposed groups. The authors also assessed the levels of oxidative stress biomarkers in the plasma by EPR spectroscopy and found high levels in the rats that were exposed in postnatal life, with the elevation being more pronounced in the double-hit model. Of note, no changes were observed in the in utero only exposed rats. Extracellular vesicle (EV) content was also evaluated- which are small membrane-enclosed vesicles that are released by many cells including endothelial cells and have been shown to elevate as a consequence of e-cigarette exposure leading to vascular dysfunction- in both adult and maternal exposures [66, 81, 82]. In the present study, EVs were shown to be increased in all e-cigarette-exposed groups, but again were more heightened in the double-hit model, which is expected as there is typically an association between oxidative stress and increased EV expression [81]. Collectively, these data indicate that MCA reactivity effects and oxidative stress are exacerbated in the double-hit model. While this study was not designed to evaluate the mechanism, it seems to suggest that epigenetic changes could play a role, with reactive oxygen species appearing to be a key contributor that creates the primary source of damage, followed by secondary contributors. Nonetheless, further mechanistic studies are clearly needed.

Some studies examined the effects of in utero e-cigarette exposure in conjunction with high-fat diet (HFD), thereby representing two common modifiable lifestyles in pregnancy. Studies have shown that maternal obesity elevates the risk of developing CVD in the offspring [83–85], and in utero exposure to HFD can result in alterations of gene expression, potentially leading to the development of metabolic disorders later in the offsprings’ life [86]. In a laboratory-based experiment, pregnant mice were exposed to HFD and e-cigarette vape to study the impact this combination had on neonatal heart [83]. At postnatal day 3 and 14, significant increase in cardiomyocyte (CM) apoptosis was observed in HFD plus e-cigarette-exposed mice, relative to the controls. Next, assessment of ventricular myofibrillar structure documented cytoplasmic and nuclear abnormalities, including shrunken nuclei, chromatin condensation/fragmentation, convoluted nuclear membranes, and enlarged mitochondria, as well as myofibrillar thinning, in the HFD plus e-cigarette-exposed mice. It was further shown that the increased CM apoptosis in the HFD plus e-cigarette-exposed pups was associated with inactivation of AMPK and enhanced oxidative stress markers (4-HNE; lipid peroxidation product). Finally, BAX (a pro-apoptotic protein) was elevated, whereas BCL-2 (an anti-apoptotic protein) was found to be decreased at postnatal day 14. The ventricular perturbation of BAX/BCL-2 ratio was further evidenced by an increase in caspase-3 activity. In summary, this study suggests that in utero e-cigarette exposure combined with HFD results in neonatal cardiac dysfunction. Of note, this study lacks a no-nicotine control e-cigarette vape group, which the authors note as a potential topic for future work.

Given that thrombosis is the main mechanism of occlusive CVD and platelets are considered key cells in this process, our lab explored the impact of in utero e-cigarette exposure in this context. In our yet to be published studies, we exposed C57 mice to e-cigarette aerosol (18-mg/ml nicotine and a menthol flavor) one week before mating and throughout pregnancy until mice gave birth. Strikingly, we found that in utero e-cigarette exposure enhanced the risk of thrombosis in the offspring mice as assessed by the FeCl_3_ carotid artery injury-induced thrombosis model. It was also found to affect hemostasis as the bleeding time was shortened, as measured by the tail bleeding time assay. In terms of a potential mechanism for these effects, we found that the in utero e-cigarette exposure enhances platelet function, including aggregation, granule secretion, and activation of the integrin αIIbβ3. Furthermore, the platelet transcriptome was also altered in the offspring mice, which suggests epigenetic changes.

**Fig. 1:**
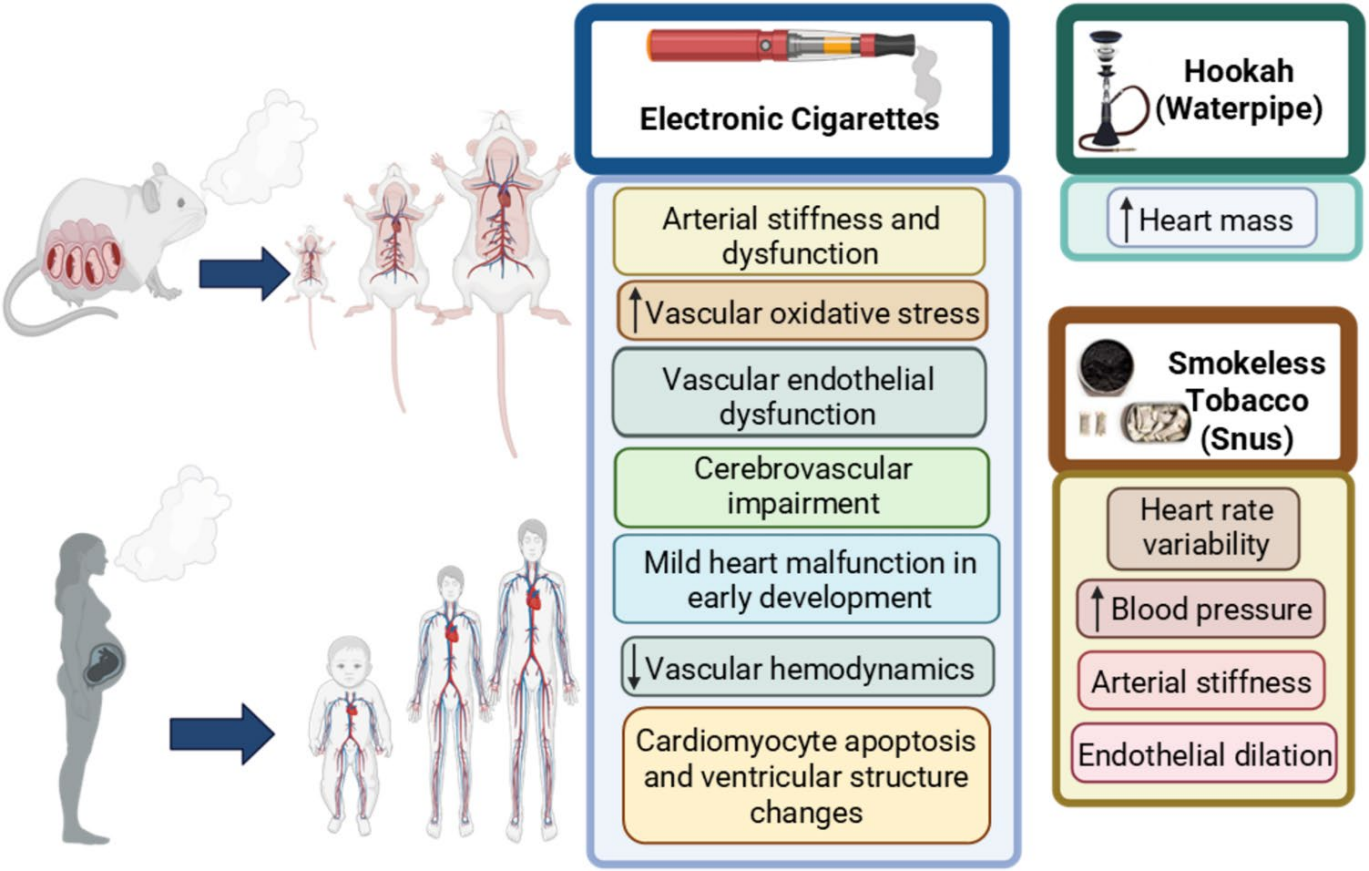
Summary of cardiovascular health impacts in the offspring of maternal novel tobacco products (e-cigarettes, hookah, and smokeless tobacco) users

Collectively, studies from animal models demonstrated that the use of e-cigarettes during pregnancy is not safe and does lead to negative cardiovascular outcomes in the offspring (Fig. 1). Due to the variety of experimental settings across these studies, the flavoring components, presence or absence of nicotine, human lifestyle issues (i.e., concomitant use of other products), and genetic variability, these studies may not strictly mimic real-life usage in humans. However, they do provide experimental evidence that should lay down the foundation for future work and inform mechanistic studies into the detrimental impact of maternal e-cigarette exposure on the cardiovascular health of the offspring that could linger into adult life.

### Hookah and Electronic Hookah

The traditional hookah device (also known as waterpipe, shisha, narghile, argileh, or hubble-bubble) has become very popular globally [87], including among youth and pregnant women [37, 88]. This device comprises a traditional waterpipe head on which flavored tobacco is placed and heated as it passes through water before being inhaled by the user. Limited literature exists concerning the effect of maternal/in utero hookah exposure on the cardiovascular function of the unborn fetus and the offspring, which includes from childhood to adult life. In one study, Wistar female rats were exposed to hookah smoke one day before mating and continued during pregnancy until birth employing the Beirut puffing protocol (171 puffs of 2.6-s duration with 17-s interval) [89]. The results revealed that the exposed rats had a reduced number of pups born relative to their control counterpart, but no differences in gender ratio were observed. Concerning their cardiovascular effects, the heart-to-body weight ratio was higher in the males’ offspring (Fig. 1) compared to the fresh air-exposed controls, suggesting a negative effect on the heart muscles, which could lead to hypertrophic cardiomyopathy [89]. Nitrate levels (a marker of NO) and catalase activity (antioxidant enzyme) were also shown to be enhanced in cardiac tissue of the in utero hookah-exposed offspring. However, other oxidative stress markers such as superoxide dismutase, glutathione peroxidase, and thiobarbituric acid reactive substances were not impacted. Notably, histologic changes in the heart of the exposed rats were not assessed, albeit needed to explain the increased heart mass [89]; thus, additional studies are needed.

Another (more recent) hookah derivative, known as the electronic hookah (e-hookah), was introduced in 2014 and marketed as a ‘healthier alternative’ to the traditional hookah [90, 91]. This device has been grossly understudied and virtually nothing is known about its in utero effects on the cardiovascular function. Nevertheless, in our unpublished data, we were able to show for the first time that in utero/maternal exposure to e-hookah has the capacity to increase the risk of thrombotic CVD in the offspring mice. In terms of the mechanism behind this phenotype, we obtained evidence that this exposure renders platelets hyperactive in nature and more prone to form clots. Consistent with these findings, we documented that direct exposure of mice to both electronic and traditional hookah had similar effect on platelets, which should serve as a template for further research [92, 93].

While these studies suggest a potential negative impact of maternal exposure to hookah and e-hookah on the cardiovascular health of offspring, more comprehensive studies using animal models are needed to mechanistically address their impact on the heart, vessels, and other CVDs.

### Smokeless Tobacco (ST)

Another popular novel tobacco product is smokeless tobacco (ST), which is best described as tobacco that can be delivered in different forms, aside from burning or smoking. Hence, they can be chewed, sucked, or sniffed when ground to powder form [94]. For example, Swedish snus (a Swedish tobacco product) is a type of moist ST that is packaged in small bags for oral use only. Besides tobacco-containing products, modern oral nicotine pouches (ONPs), a tobacco-free product that delivers nicotine orally, have also become popular [95]. While studies on in utero exposure to smokeless tobacco are very limited, a very recent study was carried out in 2024, in which participants where co-opted from a previous study of in utero smokeless tobacco exposure [96]. In this study, pregnant women who were at 6–12 weeks of gestational age were “signed-in” and their offspring were brought in for investigation at age of 5–6 years. Exposed kids were defined as those whose moms had greater than 48 mg of nicotine daily during pregnancy, while the control kids had no exposure. The results showed that children whose mothers chewed ST during pregnancy developed arterial stiffness (Fig. 1) even as early as 5–6 years of age [97], which in itself, is a risk factor for developing CVD. Also, ST-exposed children showed higher systolic (BP) readings. In a similar study using Swedish snus, the cardiovascular effect of snus was tested in exposed children aged 5–6 years. These kids were found to have a higher systolic BP and increased heart rate variability (Fig. 1) compared to unexposed kids [98]. The mechanism, though not fully understood, seems to involve interplay between the renin–angiotensin–aldosterone pathway and the autonomic nervous system [99], which may affect BP. As for the modern ONPs, which entered the US market in 2016, they are tobacco free and deliver nicotine orally when placed between the gum and lip [91]. Their popularity is increasing, particularly among vulnerable populations, due to their marketing as ‘tobacco free,’ as well as their ease of use and enticing design [93, 94]. While initial evidence suggests ONPs may deliver fewer harmful substances and are considered less harmful than other ST products due to the absence of tobacco [92], more research is needed to fully understand their long-term effects, including under in utero exposure conditions. Given their non-combustible and tobacco-free profile, the potential consideration of pregnant women switching to ONPs to quit smoking [100] necessitates a thorough evaluation of their safety for both the mother and the developing fetus.

### Maternal/In Utero Novel Tobacco Products Exposure: Safety Misperceptions and Gap in Knowledge

As indicated before, a major reason for the upsurge in the usage of NTPs among youth and pregnant women is the misperception regarding their safety profile. In line with this notion, a large number of women who smoked before pregnancy switched to e-cigarettes, and other products because of this belief [101]. Many of these products are perceived to be of ‘reduced harm/ no harm’ on health. This belief is also connected to the fact that these products are thought to be ‘emission free’ when compared to cigarettes, i.e., devoid of any toxicants that could harm the body [102]. However, it is now well established that these products are not emission free as originally advertised, as they emit high amounts of toxicants [23, 103, 104]. While e-cigarettes do not combust/burn tobacco as regular cigarettes, they do heat an e-liquid that is mainly composed of nicotine, PG/VG, and flavors, which has the potential to generate significant amounts of potentially harmful toxicants [23, 105]. For instance, PG was found to produce acrolein when heated [106], and other carbonyl compounds formed as a result of the heating process include formaldehyde and acetaldehyde agents [107], which are known carcinogens. Additionally, the evidence of the impact of flavors, which are among the major contributors to the skyrocketing use of these devices, on health is growing [108]. Thus, it is of high importance to consider the role of these constituents when investigating e-cigarette safety, especially under maternal*/*in utero exposure conditions.

In terms of hookah/waterpipes, users argue that it is safer than cigarettes because the smoke passes through water ‘filters’ off any toxins [109]. Others argue that the smoke is not inhaled, but kept in the mouth, thereby preventing nicotine inhalation. However, nicotine whas been shown to be easily absorbed through the mouth into the systemic circulation [109]. Additional factors that have contributed to their popularity include (but to limited to) appealing flavors, accessibility, social acceptance, peer pressure, and curiosity [101, 110, 111]. Indeed, waterpipe smoking is viewed as more fashionable than smoking cigarettes among women [111]. Strikingly, and as indicated before, the newer invention/e-hookah, which combines features of both hookah and e-cigarettes, has become very popular [112]. Of note, this device does use the traditional hookah apparatus, in which smoke is filtered through water before reaching the user. However, the regular head used in hookah is replaced with an electronic head in which the e-liquid is heated instead of tobacco burning [112, 113]. The safety of these devices during pregnancy is not well established and needs further studies using standardized animal models, all while considering the role of flavors, device used, etc.

Regarding smokeless tobacco, it is widely accepted as a safer alternative to traditional cigarettes; however, this misperception is mainly fueled by the notion that a smoke-free device is synonymous to having a ‘less harmful’ product [114]. On the contrary, evidence-based research has shown that smokeless tobacco contains over 30 harmful substances that promote the pathogenesis of CVDs and diabetes, among others [115, 116]. Although some of these substances have been shown to be teratogenic in nature [117], still there remains some unanswered questions regarding ST use in pregnancy. For instance, it is unknown if a particular gestational window for exposure is critical, thereby potentiating detrimental health outcomes in the offspring. Also, bearing in mind that these products can be administered in various ways, further investigation is necessary to determine if the route of administration as well as product variability could impact the bioavailability of the ST to the offspring in utero [118].

Concerning other novel tobacco products, such as heat-not-burn (HNB), there has been no studies conducted in the area of in utero use and cardiovascular function. However, considering the growing use of these devices and the unique design in which tobacco is heated not burned, there is an urgent need to characterize their cardiovascular safety during early critical development period and later in life. In fact, a recent HNB device, IQOS, is rapidly gaining popularity among young adults’ following its recent introduction into the market in 2019 [119]. This increase could be attributed, in part, to awareness that has been made through tobacco cessation/reduction marketing strategies that strongly emphasize lung injuries associated with e-cigarette vaping, causing the youth to exploit other options for nicotine consumption [120]. These products can deliver flavored aerosols containing nicotine, without combustion of the tobacco. They achieve this by heating tobacco at low temperatures (< 600°C) compared to traditional cigarettes, hence fueling the belief that it is a safer alternative [121]. In line with this thought, pregnant women often switch to these types of products and consider them to be a ‘safer’ smoking cessation option [121]. Though there has yet to be a study that highlights the impacts of HNB products on pregnant women or the offspring during any stage of life, these products nonetheless contain nicotine which has been shown to cross the placenta and negatively impact fetal growth [118]. Taken together, these issues should underscore the need for additional research in this regard.

## Conclusion

Tobacco companies have exploited the already established disadvantages of cigarette smoking, especially in pregnancy to ‘drive’ the popularity of NTPs. Hence, as noted earlier, the use of these products is rising at an alarming rate, among vulnerable populations, which includes pregnant women, thus impacting innocent fetuses. A major challenge in sensitizing the public to the merits of using these products is the lack of adequate research; which should also define the mechanisms/pathways that underly their effects, among other issues (e.g., role of flavor). The significant variability between individual products and users behavior raises a series of questions specific to their unique features and diversity of user behavior. This underscores the complexity of this topic and the limited information in the current literature. E-cigarettes are obviously the most studied product within this group, but still, there is a lot that is understudied, especially in the context of maternal and in utero exposures, as well as other CVDs. Thus, there should be concerted efforts to shed more light onto these issues, as existing literature is full of knowledge gaps regarding the cardiovascular risk of using these products and defining the mechanisms behind their effects; which is of critical importance since smoking/vaping is known to be the single most preventable risk factor for developing CVDs. Taken together, this review summarized the literature (Table 1), albeit very limited, and mainly provided by animal models, which clearly demonstrated the negative cardiovascular consequences of using these devices in pregnancy on the health of the offspring and underscored the need for more aggressive research in this area.

## Data Availability

No datasets were generated or analyzed during the current study.
